# Precision medicine in neurodegeneration: the IHI-PROMINENT project

**DOI:** 10.3389/fneur.2023.1175922

**Published:** 2023-08-02

**Authors:** Ashley Tate, Marc Suárez-Calvet, Mats Ekelund, Sven Eriksson, Maria Eriksdotter, Wiesje M. Van Der Flier, Jean Georges, Miia Kivipelto, Milica G. Kramberger, Peter Lindgren, Juan Domingo Gispert López, Jyrki Lötjönen, Sofie Persson, Sandra Pla, Alina Solomon, Lennart Thurfjell, Anders Wimo, Bengt Winblad, Linus Jönsson, on behalf of the PROMINENT consortium

**Affiliations:** ^1^Department of Neurobiology, Care Sciences and Society, Karolinska Institutet, Stockholm, Sweden; ^2^Barcelonaβeta Brain Research Center, Pasqual Maragall Foundation, Barcelona, Spain; ^3^IMIM (Hospital del Mar Medical Research Institute), Barcelona, Spain; ^4^Centro de Investigación Biomédica en Red de Fragilidad y Envejecimiento Saludable (CIBERFES), Madrid, Spain; ^5^Servei de Neurologia, Hospital del Mar, Barcelona, Spain; ^6^BioArctic AB, Stockholm, Sweden; ^7^Karolinska University Hospital, Theme Inflammation and Aging, Stockholm, Sweden; ^8^Alzheimer Center Amsterdam, Neurology, Vrije Universiteit Amsterdam, Amsterdam UMC, location VUmc, Amsterdam, Netherlands; ^9^Amsterdam Neuroscience, Neurodegeneration, Amsterdam, Netherlands; ^10^Alzheimer Europe, Luxembourg, Luxembourg; ^11^University Medical Center Ljubljana and Medical Faculty, University of Ljubljana, Ljubljana, Slovenia; ^12^IHE, The Swedish Institute for Health Economics, Lund, Sweden; ^13^Universitat Pomepu Fabra, Barcelona, Spain; ^14^Centro de Investigación Biomédica en Red de Bioingeniería, Biomateriales y Nanomedicina (CIBER-BBN), Madrid, Spain; ^15^Combinostics Oy, Tampere, Finland; ^16^Synapse Research Management Partners SL, Madrid, Spain; ^17^Institute of Clinical Medicine and Public Health and Clinical Nutrition, University of Eastern Finland, Kuopio, Finland

**Keywords:** Alzheimer’s disease, dementia, precision medicine, biomarkers, clinical decision support

## Abstract

Neurodegenerative diseases are one of the most important contributors to morbidity and mortality in the elderly. In Europe, over 14 million people are currently living with dementia, at a cost of over 400 billion EUR annually. Recent advances in diagnostics and approval for new pharmaceutical treatments for Alzheimer’s disease (AD), the most common etiology of dementia, heralds the beginning of precision medicine in this field. However, their implementation will challenge an already over-burdened healthcare systems. There is a need for innovative digital solutions that can drive the related clinical pathways and optimize and personalize care delivery. Public-private partnerships are ideal vehicles to tackle these challenges. Here we describe the Innovative Health Initiative (IHI) public-private partnership project PROMINENT that has been initiated by connecting leading dementia researchers, medical professionals, dementia patients and their care partners with the latest innovative health technologies using a precision medicine based digital platform. The project builds upon the knowledge and already implemented digital tools from several collaborative initiatives that address new models for early detection, diagnosis, and monitoring of AD and other neurodegenerative disorders. The project aims to provide support to improvement efforts to each aspect of the care pathway including diagnosis, prognosis, treatment, and data collection for real world evidence and cost effectiveness studies. Ultimately the PROMINENT project is expected to lead to cost-effective care and improved health outcomes.

## 1. Introduction

### 1.1. Aim

The aim of this project is to create a platform for precision medicine in the diagnosis and treatment of neurodegenerative disease and comorbidities. This digital platform will integrate multi-modal diagnostic data to generate personalized prediction of patient relevant outcomes as well as evidence-based recommendations for clinical management. The platform will also support the implementation of new diagnostic and therapeutic innovations and provide required evidence on safety, efficacy, and cost-effectiveness for relevant stakeholders.

Expected key impacts of the project include:

1. increased precision in diagnosis, prognosis, and management of patients with (suspected) neurodegenerative disorders and comorbidities,
2. optimal introduction and use of new health technologies, such as disease-modifying therapies (DMT), leading to improved patient outcomes, and
3. empowerment of patients and caregivers by through person-centric health care decisions, leading to improved adherence and reduced inequalities in access to care

### 1.2. The PROMINENT consortium

PROMINENT represents the first project funded by the Innovative Health Initiative (IHI), an extension of the Innovative Medicine Initiative. With the ultimate aim of fostering the translation of health research and innovation into tangible progress for patients and society, the IHI is a collaboration between the European Union and industry associations representing the healthcare sector: COCIR (medical imaging, radiotherapy, health ICT and electromedical industries); EFPIA, including Vaccines Europe (pharmaceutical industry and vaccine industry); EuropaBio (biotechnology industry); and MedTech Europe (medical technology industry). The IHI funds collaborative, innovative, and interdisciplinary projects which have a patient-centered approach. The first call for proposals for projects related to innovation in cancer, neurodegenerative diseases, and health data was issued on June 2022. PROMINENT was notified of their award of grant funding in November, 2022.

The PROMINENT consortium is comprised of 13 universities, research institutes, hospitals, companies, and patient groups, with teams highly specialized in medical technology development and dementia care and research. Together, we have harnessed our already existing digital health tools, prediction models, comprehensive data sources, and expertise to push the dementia care to new frontiers. Our ambition is firmly based in our currently running projects, and while our expectations are high the steps forward are merely incremental from previously awarded grants and projects.

### 1.3. Background

AD is a neurodegenerative disease, and the etiology behind 50–70% of all cases of dementia (1). The prevalence of symptomatic AD (prodromal, i.e., pre-dementia, AD or AD dementia) in Europe has been estimated at 22.1 million, while the estimated number of cognitively unimpaired persons with abnormal AD biomarkers is as high as 53.2 million (2). Due to an ageing population, dementia prevalence is expected to nearly double over the coming three decades, bringing enormous challenges for health and social care systems (3).

There is rapid development in the diagnosis and treatment of AD and other neurodegenerative disorders (4), across plasma-based biomarkers (5), pharmacological treatments and non-pharmacological prevention strategies (6). Thus, avenues are emerging for earlier and more accurate detection and diagnosis, however the implementation of these advancements is challenging. There is opportunity to improve on current care practices, which often include late, unspecific diagnosis and mainly palliative care provision, in favor of precise diagnostics and early treatment – a transformation similar to what has been seen in oncology over past decades (1). However, ensuring appropriate use and access to novel diagnostics and treatment is problematic, primarily due to lack of resources and available expertise. Limited capacity in the primary care settings and, occasionally, in specialist memory clinics can also lead to the prioritization of use in younger patients without significant comorbidities.

Implementing new technologies and latest treatments, such as emerging disease-modifying drugs lecanemab and donanemab, in routine care will be challenging, and the availability of specialists in neurology and geriatrics is a constraining factor in many European countries (7). Given the high prevalence of subjective cognitive decline (SCD), effective selection mechanisms are needed to identify those who are likely to have underlying AD pathology in a primary care or community setting. Therefore, there is a need for a clinical decision support system that can assist specialists as well as non-specialists with the interpretation of complex, multi-modal diagnostic data and provide accurate diagnostic and prognostic predictions derived from reference populations.

## 2. The PROMINENT digital platform

### 2.1. Overview

**Figure 1:**
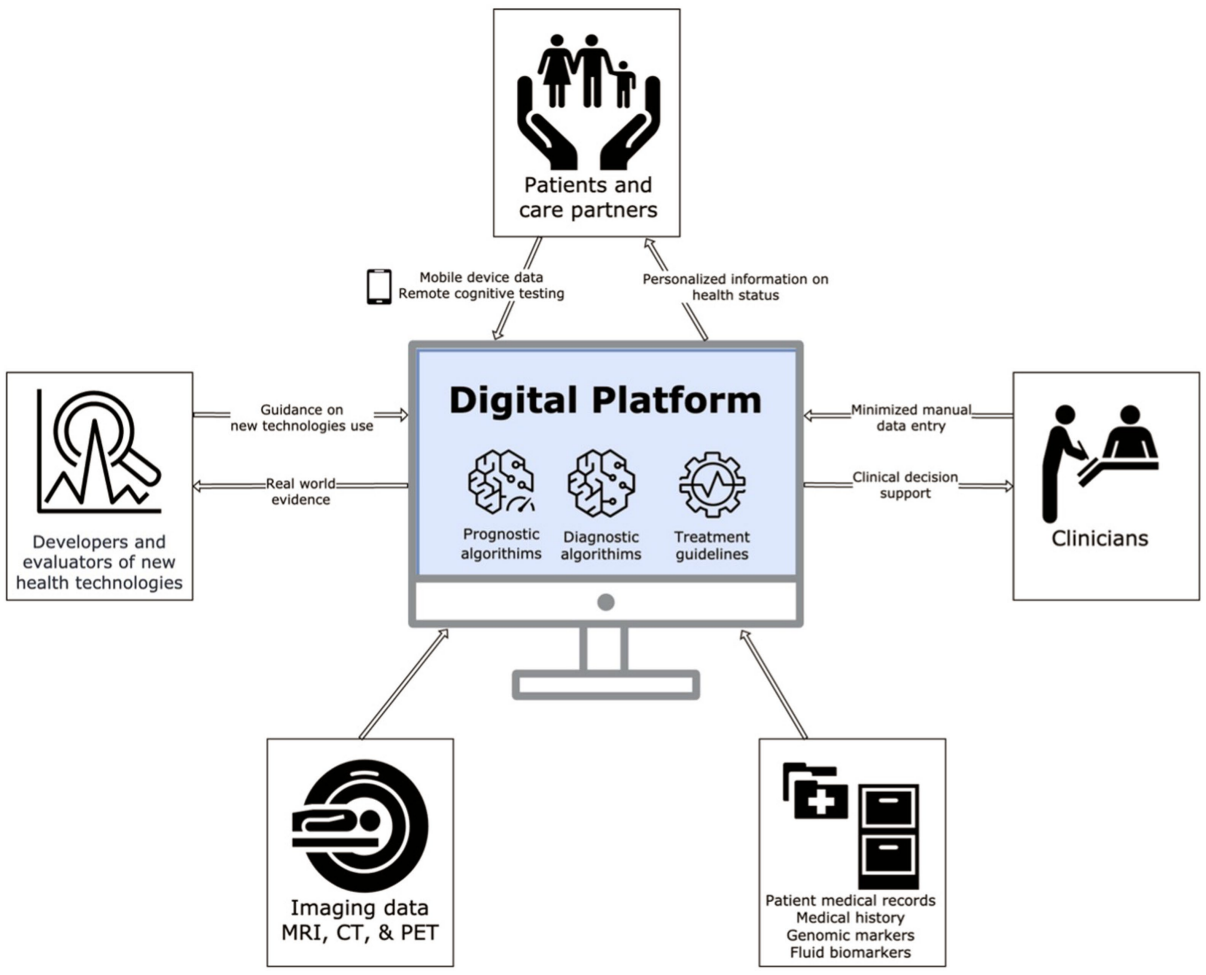
This figure shows what information is fed into and obtained from the digital platform, indicated by the arrow direction. The digital platform itself relies on an underpinning of prognostic and diagnostic algorithms and treatment guidelines. Adapted with permission from Combinostics.

The digital platform that will be developed in PROMINENT is visualized in Figure 1. At the core of the platform are prediction models trained on large, representative datasets, capable of producing predictions of the correct diagnosis, future course of disease and care needs based on a wide range of factors including demographics, clinical history, cognitive tests, diagnostic imaging, fluid biomarkers and genetics. These predictions are combined with data on performance and cost of diagnostic tests, to produce optimized diagnostic algorithms. The platform will also incorporate updated guidelines on the use of relevant therapies, which will be leveraged to produce individualized eligibility assessments.

The platform will give personalized, timely and accurate information about the current disease state, prognosis, and potential benefits and risks with novel therapies. The primary target user group for the platform is clinicians engaged in the care of patients with neurodegenerative disorders. Based on available information on the individual patients being examined, the platform will provide guidance to clinicians on the likelihood of differential diagnoses, the likely future prognosis, and recommended next steps in the diagnostic process. The system will also summarize available information about the potential benefit and risk of disease-modifying therapy, tailored to the individual patient, and provide an assessment of the eligibility for treatment.

Patient and caregiver engagement will be promoted through individualized, lay-language information about diagnosis, prognosis and treatment recommendations. The system will provide longitudinal predictions of the likely course of disease in terms of cognitive decline and major events of relevance to patients and care partners such as loss of independence in key functions, institutionalization, and mortality (8). Visualizing this information in a manner that is understandable and meaningful is paramount, so patients and care partners will be closely engaged in the design of the system and outputs. These materials will be developed in dialog with patient and caregiver representatives. Such information materials may greatly improve communication provided by the physician and provide a concrete document patients and care partners can take with them from the visit. The materials will be developed in close collaboration/co-design with care partners and patients, enabled by public engagement mechanisms developed by Alzheimer Europe. We will also explore the feasibility of including evidence-based recommendations for patients and care partners relating to non-pharmacological prevention, availability of support services and similar relevant information, based on current disease status and individualized risk predictions.

Further, by prospectively capturing data on patients treated with disease-modifying therapies, the system is expected to produce valuable information for the assessment of the response to therapy, adverse events experienced, and the overall value and cost-effectiveness delivered by these therapies. This will enable health economic evaluation of diagnostic and therapeutic strategies, and implementation decision rules to guide cost-effective use of novel technologies.

### 2.2. Developing the digital platform

The PROMINENT digital platform will be developed from an existing, commercially available system for AI-based diagnostic imaging analysis and CDSS: Combinostics cNeuro. This system is already commercially available and used in hospital settings across Europe and the United States. cNeuro is a cloud-based tool including two components: cMRI and cDSI. cMRI is an AI-based tool for quantification of MRI-brain images and is mainly used in dementia and multiple sclerosis (9). Findings are summarized in PDF-reports that contain information about atrophy and lesions, which assists radiologists in making more detailed and consistent reports in shorter time.

The second component, the Disease State Index or cDSI, assists clinicians with the differential diagnosis of patients with suspected dementia. cDSI combines imaging information (output from cMRI) with other manually entered patient data such as demographics, CSF-biomarkers, and cognitive test scores. Then, the patient’s data profiles are compared to a database with data from previously diagnosed patients with a known outcome. cDSI provides information about differential diagnostics and prediction of progression, i.e., the disease state index. cNeuro has been validated using both retrospective and prospective data. In a comprehensive prospective study with 800 patients, the tool was found to increase clinicians’ confidence in making early diagnostic decisions (10). A screenshot of the tool is shown in Supplementary Figure 1.

The existing systems will be developed into an open, interoperable platform (Figure 1) capable of interacting with a wide range of other systems to acquire data and deliver outputs. Key advancements of the PROMINENT digital platform compared to existing systems will include:

- Interoperability with EHR and other systems, reducing or eliminating the need for manual data entry, improving functionality and usability.
- Enhanced imaging analysis capabilities, including ARIA E/H detection.
- Interactivity with patients and care partners, including development of a web solution and/or mobile app for remote assessment of cognition and other functions, and individualized outputs from the platform designed to facilitate communication and shared decision making.
- Prediction models validated in routine care patient populations, accounting for comorbidities and incorporating novel blood-based biomarkers in addition to other predictors
- Clinical decision support including recommendations of optimal, cost-effective diagnostic pathways, the probability of diagnostic accuracy, and costs of individual test components.
- A tool for guiding clinicians on the optimal use of novel interventions such as DMT for AD and generating real-world evidence (RWE) on the actual usage, safety, effectiveness, and cost-effectiveness of these technologies in routine care.

### 2.3. Prediction models and diagnostic algorithms

In PROMINENT we aim to consolidate (rather than replicate) the vast body of research into prediction models for AD diagnosis and prognosis. This work will focus on predictions within symptomatic populations (SCD, mild cognitive impairment [MCI], or dementia), as this is currently the clinically most relevant population for diagnosis and potential treatment with DMT (11). We will first conduct an updated systematic review of different categories of multimodal prediction models and select models of potential clinical usefulness. We plan to specifically examine models related to differential dementia disease diagnosis and time until disease progression. Next, we will implement and expand on selected models using machine learning where the models are trained with the diverse datasets available to the consortium. Table 1 presents an overview of the outcomes that will be targeted in the modeling, and the range of predictors that will be included. Additional outcomes may be included based on consultation with patients, caregivers, and medical professionals through surveys.

**Table 1:** Outcomes and tentative predictors categories for prediction modelling.

Outcomes	Classes of predictors
*Underlying etiology* Differential diagnosis: probability that the patient currently has one of several neurodegenerative and neurovascular disorders, or does not have any neurodegenerative disorder. Standard of truth will be the reference diagnosis at end of follow-up based on clinical and biomarker criteria.	Co-morbidity profile Cognitive function Blood biomarkers (p-tau, NfL, GFAP) CSF biomarkers (amyloid, tau, p-tau) Diagnostic imaging (MRI, PET) Genetic markers (e.g., APOE) Demographics Concomitant medication Socioeconomic factors
*Future health outcomes* Probability of disease progression in terms of disease state: subjective cognitive impairment (SCD) to mild cognitive impairment (MCI) to dementia, over time Longitudinal decline in cognitive function scores (e.g., MMSE, MoCA, neuropsychological test batteries) and other clinical scales (ADL, NPI) Institutionalization and other changes in care setting and provision of support services Hospitalizations, health care resource utilization, and costs Mortality	

Importantly, we will explore the impact of comorbidities on diagnostic and prognostic accuracy. Specific focus will also be placed on integrating data from blood-based biomarkers into prediction models and diagnostic algorithms. This includes (1) predicting the likely outcome of the blood-based biomarker given a set of known patient characteristics, (2) predicting the results of downstream diagnostic investigations in patients who test positive vs. negative on the blood-based biomarker, (3) including the result of the biomarker test in predictive models of future disease progression and clinical events.

Based on these prediction models, diagnostic algorithms will be developed that in a stepwise fashion estimate the expected outcomes of the next potential diagnostic test, and identifies the sequence of tests that produces the optimal overall diagnostic performance and cost.

Finally, we will validate the models in data reflecting routine clinical practice in unselected populations across different care settings and evaluate performance outside of the datasets used for developing the models.

### 2.4. Datasets for prediction model development and validation

The consortium possesses eight large datasets (registries and cohorts) with a combined total sample of over 128,000 participants that cover the full spectrum of AD from preclinical stages, through to dementia and end-of-life institutional care. Supplementary Table 1 provides a description of the included datasets. As the datasets represents currently on-going studies, the PROMINENT consortium will benefit from existing knowledge from each of the data holders on the strengths and weaknesses of each dataset. Moreover, the collaboration ensures that scientific efforts within each register and data source will be harmonized.

Further, the PROMINENT consortium will seek to collaborate with other projects across European and national initiatives, such as (but not limited to) MOPEAD (12), PRODEMOS (13), EUROFINGER (14), ROADMAP (15), NEURONET (16), PREDEM (17), EPND (18), and ABOARD (19), as well as the recently announced IHI projects AD-RIDDLE and PREDICTOM.

### 2.5. Security considerations

When developing the predictive models, we aim to minimize the transfer of data through the use of federated architecture. The system will not disclose patient level information nor will the models output any patient identifying information. An independent ethics advisor will be appointed to monitor any issues that may arise.

## 3. Evaluation and validation of the digital platform

Two prospective studies will soon begin to (1) *evaluate* how well the decision support system provides relevant, actionable information to clinicians, patients, and care partners, and (2) generate *validation* data on the accuracy of the diagnostic and prognostic estimate of the system, compared with clinical reference standards and actual outcomes.

The evaluation study will be conducted by digital surveys to patients and care partners, before and after using the system. Structured interviews will be conducted with clinicians, patients, and care partners at each site to obtain detailed feedback, identify issues and opportunities for improving the system and user experience.

The validation study will be conducted as a prospective, single-arm study where the intervention (CDSS) is received by all participants. Study participants will include patients attending regular initial visits for a suspected cognitive disorder at participating specialist clinics. Inclusion criteria will be broad to reflect the patient population seen under routine care conditions. Subjects who contributed to data used in the training of the predictive algorithms will not be included.

The primary endpoint is the diagnostic accuracy of the CDSS, and will be measured by comparing the system’s output with the clinical diagnosis at baseline and after 24 months of follow-up, as rated by an independent panel of specialists. The accuracy of predictions of disease progression will similarly be assessed by comparing system predictions with actual disease progression (e.g., change in MMSE scores from baseline) at 24 months of follow-up. When possible, data will be collected directly through the CDSS. Confidence in diagnosis will be assessed through visual analog scales administered by questionnaire to clinicians before and after accessing the system. The targeted sample size is 125–150 patients per site, for a total sample size of around 800 patients. The instrument used for assessment of cognitive status (MMSE or MoCA), will be determined through local practices as well as licensing conditions for the instrument to ensure data availability.

## 4. Applying the platform to support introduction of new technologies

At the time of writing, the recent approval of lecanemab and positive trial results for donanemab spell an exciting advancement for the treatment of dementia. However, the process of integrating the treatments into the healthcare system is complex. Thus, there is an increased need for support across the clinical implementation process. The PROMINENT digital platform can make important contributions by enabling generation of RWE and evaluation of new interventions as well as their pricing and reimbursement.

Starting within the clinic, the platform can identify eligible participants, calculate risks and benefits, and provide recommendations on how to initiate treatment at the individual level. The system will do so through comparing patient characteristics with eligibility criteria and evidence from clinical trials or other studies. In this way, the platform will impact treatment strategy and triaging. Once treatment is initiated, the system will provide a framework for consistent follow-up of treatment effectiveness and monitoring of side-effects, in line with what is needed to generate RWE data and health technology assessments (HTA). The main task of reimbursement agencies and HTA bodies is to ensure appropriate, safe, and cost-effective use of innovative therapies.

The generated data from the digital platform will contain de-identified patient-level data that is summarized, and then aggregated across health care providers. Based on this aggregated data, pre-defined analyses and reports are generated that answer key questions about the uptake, usage, impact, and outcomes with the new technology of interest. The focus will be on metrics of interest and relevance for reimbursement agencies and HTA bodies, to allow clinicians to fulfill requirements on follow-up and data collection with minimum administrative burden.

By generating RWE data, the platform will assist pharmaceutical companies in developing new interventions for neurodegenerative disorders by likely reducing the cost and burden of stand-alone phase IV clinical studies and increase the accessibility of healthcare databases. Thus, the successful implementation of the RWE module may contribute to speedier (re)assessment and follow-up by agencies benefiting both patients and innovators.

We will develop a set of specifications for the HTA module based on input from agencies. A standardized analysis protocol will be developed to obtain the metrics of interest relating to safety, efficacy, and cost-effectiveness. The protocol will serve as a template for the implementation of specific technologies and will be developed in consultation with clinicians, health technology assessment bodies, patients, and care partners.

## 5. Project limitations and potential obstacles

Based on the current study design there are a few limitations and obstacles that we must take into consideration. First, the harmonization of the different data sources represent a key hurdle, as available data types differ across each source. Next, there could be some difficulties in implementation of the digital platform for countries which do not make extensive use of EHR, thus limiting the accuracy in comparison to countries with complete EHR coverage. Finally, the PROMINENT digital platform will primarily be targeted to memory clinics, although we hope to extend its use to primary care in the future.

## 6. Potential impact of PROMINENT

### 6.1. Broader impact

#### 6.1.1. Understanding comorbidities

Patients seen in routine care with a suspected cognitive disorder often have comorbid illnesses, such as cardiovascular disorders, type 2 diabetes, and psychiatric disorders (20). Comorbidities can have a decisive influence on neurodegenerative disease diagnostic investigations and the interpretation of their results, e.g., reduced renal function can influence cut-off values on plasma biomarkers which changes the interpretation of test results (21). Additionally, the presence of comorbidities can also impact the initiation of treatment. The appropriate use recommendations for aducanumab, the first therapy to obtain regulatory approval in the United States, do not recommend treatment for patients with comorbid neurological or psychiatric conditions, or any poorly controlled or severe medical illness until the condition is ‘managed and stable’ (22). This guidance is nonspecific with a limited evidence base since patients with significant comorbidities were excluded from the clinical trials of this therapy. Despite the clear impact of comorbidities on health outcomes, research has been hampered in part due to small study sizes. The development and utilization of the PROMINENT digital platform can provide important contributions to this field through the utilization of our data.

#### 6.1.2. Overarching impact

The PROMINENT digital platform aims to make improvements on each aspect of the healthcare system, as well as to facilitate the cohesion of patients, care partners, clinicians, reimbursement agencies, and HTA organizations. This collaborative effort represents the incremental refinement of a digital platform already available for use in a clinical setting. Patients will directly benefit from the information provided by the platform on disease status, prognosis, and potential risks and benefits of novel therapies. Moreover, the already built in data collection feature will save clinicians time and reduce the administrative strain, while also benefiting reimbursement agencies and HTA organizations. Although, the primary target of the platform is Alzheimer’s disease (AD) and other dementia disorders, scalability is possible to other neurodegenerative diseases and beyond. While ambitious, it is our hope that this project represents a tangible first step toward a paradigm shift in the care of neurodegenerative disorders.

## Data availability statement

The original contributions presented in the study are included in the article/Supplementary material, further inquiries can be directed to the corresponding author.

## PROMINENT Consortium members

Karolinska Institutet, Stockholm, Sweden; Combinostics Oy, Tampere, Finland; Synapse Research Management Partners SL, Barcelona, Spain; Alzheimer Europe, Luxembourg, Luxembourg; Region Stockholm, Sweden; BioArctic AB, Stockholm, Sweden; Klinikum der universitaet zu Koeln, Cologne, Germany; IHE Institutet för Hälsoekonomi AB, Lund, Sweden; Barcelonaβeta Brain Research Center; Pasqual Maragall Foundation. Barcelona, Spain; CHU Hopitaux de Bordeaux, Bordeaux, France; University Medical Center, Lubljana, Slovenia; ITA-Suomen Ylipisto, Kuopio, Finland; Stichting VUMC, Amsterdam, the Netherlands.

## Author contributions

LJ and AT drafted the first version of the manuscript. All authors reviewed the manuscript and approved the final version.

## Conflict of interest

ME and SE were employed by BioArctic AB. JL and LT were employed by Combinostics Oy.

The remaining authors declare that the research was conducted in the absence of any commercial or financial relationships that could be construed as a potential conflict of interest.

## Publisher’s note

All claims expressed in this article are solely those of the authors and do not necessarily represent those of their affiliated organizations, or those of the publisher, the editors and the reviewers. Any product that may be evaluated in this article, or claim that may be made by its manufacturer, is not guaranteed or endorsed by the publisher.

## Supplementary material

Supplementary Figure 1 The figure shows the cDSI tool with the list of biomarkers available for the patient (left), the classification panel (middle), and a comparison of the patient’s disease state (yellow line) versus the distributions of AD and FTD cases (right). The tool shows that the patients data profile fits best to the distribution of previous AD cases, with FTD being the second most likely diagnosis.
