# Supplementary figures and images for: Precision medicine in neurodegeneration: the IHI-PROMINENT project
Source: Front Neurol. 2023 Aug 2;14:1175922. doi: 10.3389/fneur.2023.1175922 (PMC10433183; doi:10.3389/fneur.2023.1175922)

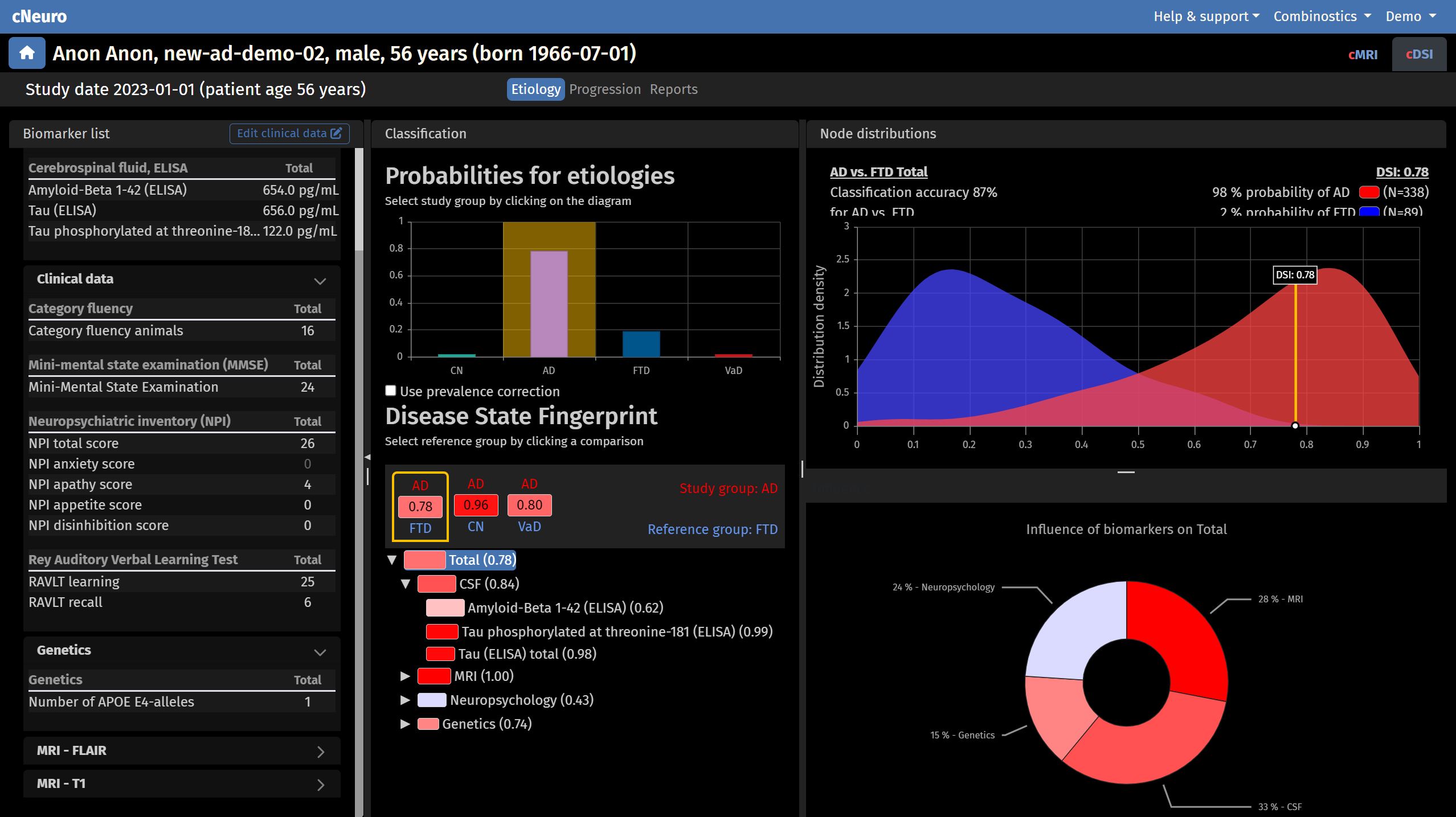

Supplement: Supplementary Figure 1 — The figure shows the cDSI tool with the list of biomarkers available for the patient (left), the classification panel (middle), and a comparison of the patient’s disease state (yellow line) versus the distributions of AD and FTD cases (right). The tool shows that the patients data profile fits best to the distribution of previous AD cases, with FTD being the second most likely diagnosis. [file Image_1.JPEG]
