# Supplementary material for: Precision medicine in neurodegeneration: the IHI-PROMINENT project
Source: Front Neurol. 2023 Aug 2;14:1175922. doi: 10.3389/fneur.2023.1175922 (PMC10433183; doi:10.3389/fneur.2023.1175922)
Supplement: Supplementary file 2 [file Table_1.pdf]

Supplemental Table 1. Overview of data sources

Data source	Country	Year established	Sample	Source	Follow-up	Data available
SveDem [23]	Sweden	2007	109,000 patients with MCI or dementia	Population	annual	☒Cognitive function<br>☐Fluid biomarkers<br>☐ Neuroimaging<br>☐Genetic<br>☒ Comorbidities<br>☒Treatment<br>☒Life factors
GEDOC [24]	Sweden	1998	>7000	Memory clinic		☒Cognitive function<br>☒Fluid biomarkers<br>☒ Neuroimaging<br>☒Genetic

						☐ Comorbidities<br>☐ Treatment<br>☐ Life factors
Kuopio Research Cohort [25]	Finland		200 participants including patients and controls	Memory clinic	annual	☒ Cognitive function<br>☒ Fluid biomarkers<br>☒ Neuroimaging<br>☐ Genetic<br>☒ Comorbidities<br>☒ Treatment<br>☐ Life factors
ALFA+ [26]	Spain		420 unimpaired participants, most children of AD patients	Research cohort	Every 3 years	☒ Cognitive function<br>☒ Fluid biomarkers<br>☒ Neuroimaging<br>☒ Genetic

						☐ Comorbidities<br>☐Treatment<br>☒Life factors
BETA-AARC [27]	Spain	2022	200 participants with SCD or MCI	Research cohort	annual	☒Cognitive function<br>☒Fluid biomarkers<br>☒ Neuroimaging<br>☐Genetic<br>☐ Comorbidities<br>☐Treatment<br>☒Life factors
DELCODE [28]	Germany	2015	1000 participants including AD patients, controls, and unimpaired with a first degree	Memory clinic	annual	☒Cognitive function<br>☒Fluid biomarkers<br>☒ Neuroimaging<br>☐Genetic

			relative with AD			☐ Comorbidities<br>☐Treatment<br>☐Life factors
MEMENTO [29]	France	2011	2323 patients with MCI or dementia	Memory clinic	annual	☒Cognitive function<br>☒Fluid biomarkers<br>☒ Neuroimaging<br>☒Genetic<br>☒ Comorbidities<br>☒Treatment<br>☒Life factors
REAL AD [30]	Sweden	2023	Collection not yet started; aims to provide a reference panel for normal cognitive tests and	Research cohort	NA	☒Cognitive function<br>☒Fluid biomarkers<br>☒ Neuroimaging<br>☐Genetic

			biomarker values			☐ Comorbidities<br>☐Treatment<br>☐Life factors
Amsterdam Dementia Cohort [31]	The Netherlands	2000	~8000	Memory clinic	annual	☒Cognitive function<br>☒Fluid biomarkers<br>☒ Neuroimaging<br>☒Genetic<br>☒ Comorbidities<br>☒Treatment<br>☒Life factors
